# Supplementary material for: Prolonged Antiretroviral Treatment Induces Adipose Tissue Remodelling Associated with Mild Inflammation in SIV-Infected Macaques
Source: Cells. 2022 Oct 2;11(19):3104. doi: 10.3390/cells11193104 (PMC9561982; doi:10.3390/cells11193104)
Supplement: Supplementary file 1 [file cells-11-03104-s001.zip › cells-1906025-supplementary.pdf]

# Supplementary Materials

**Supplementary Methods**:
qPCR reactions protocol :
We assayed 18 genes by PCR including the 2 housekeeping genes using single PCR reaction. PCR plate were designed as follow :

- 2 individuals from different group in each plate (SIV-/SIV+ or SIV-/SIV+ART+ or SIV+/SIV+ART+)
- SCAT and VAT samples of each animal on the same plate
- 8 genes of interest and PPIA expression by plate , using duplicate.
- In total, 2 plates were needed to study all of our genes of interest in each individual

| Gene name | Stability value |
| --- | --- |
| 18S | 1.262 |
| PPIA | 1.236 |
| RPLPO | 0.533 |
| 18S and PPIA | 0.527 |

**Table S1**: The various housekeeping genes' stability values, calculated using NormFinder software and 204 gene expression values.

| | SIV- | | SIV+ | | | | SIV+ART+ | | | | | |
|---|---|---|---|---|---|---|---|---|---|---|---|---|
| | Medians | IQR | Medians | IQR | Ratio /SIV- | **p-value** | Medians | IQR | Ratio /SIV- | **p-value** | Ratio /SIV+ | **p-value** |
| PPARG | 0.002386 | 0.0159 | 0.001079 | 0.0401 | 0.45 | **>0.99** | 0.0461 | 0.583 9 | 19.3 | **0.01** | 42.7 | **0.0005** |
| CEBPA | 0.006831 | 0.0149 | 0.004538 | 0.0787 | 0.66 | **>0.99** | 0.0749 | 0.994 3 | 11.0 | **0.04** | 16.5 | **0.005** |
| COL1A2 | 0.000941 | 0.0372 | 0.001863 | 0.8842 | 2.0 | **>0.99** | 0.1931 | 3.163 | 205.3 | **0.005** | 103.7 | **0.02** |
| PPARG/ COL1A2 | 2.497 | 11.40 | 0.4633 | 3.260 | 0.18 | **0.15** | 0.3175 | 1.774 | 0.12 | **0.07** | 0.69 | **>0.99** |
| HIF1A | 0.001321 | 0.0177 | 0.001203 | 0.1278 | 1.0 | **>0.99** | 0.02580 | 0.550 9 | 22.0 | **0.02** | 21.4 | **0.04** |
| CD45 | 7.841 e-007 | 1.758 e-005 | 2.054 e-007 | 0.0002 | 0.3 | **>0.99** | 3.154 e-005 | 0.002 355 | 40.2 | **0.6** | 153.6 | **0.3** |
| CD163 | 0.000656 | 0.0023 | 0.0001732 | 0.0198 | 0.3 | **>0.99** | 0.005941 | 0.163 4 | 9.06 | **0.006** | 34.3 | **0.01** |
| IFNG | 4.237e-008 | 7.692e 6007 | 1.533 e-007 | 3.065 e-006 | 3.6 | **0.8** | 5.501e-007 | 0.000 2362 | 13.0 | **0.087** | 3.6 | **0.8** |
| IL6 | 0.001727 | 00.008 222 | 0.0001180 | 0.0123 1 | 0.07 | **0.7** | 0.000883 7 | 0.033 29 | 0.5 | **>0.99** | 7.5 | **0.8** |
| TNFA | 7.841 e-007 | 5.182 e-005 | 1.743 e-007 | 0.0005 385 | 0.22 | **>0.99** | 6.260 e-005 | 0.005 880 | 80 | **>0.99** | 359.2 | **0.5** |
| ADIPO Q | 0.01853 | 0.0086 99 | 0.01973 | 0.7699 | 1.06 | **>0.99** | 0.3920 | 4.807 | 21 | **0.009** | 19.9 | **0.04** |
| LEP | 0.03921 | 0.2966 | 0.02806 | 2.456 | 0.7 | **>0.99** | 0.3373 | 5.410 | 8.6 | **0.15** | 12.0 | **0.03** |
| ADIPO Q/LEP | 0.3882 | 6.958 | 0.5548 | 3.200 | 1.43 | **0.84** | 1.261 | 2.931 | 3.25 | **0.011** | 2.3 | **0.17** |
| CCL5 | 0.000589 3 | 0.0019 32 | 0.002709 | 0.0331 9 | 4.6 | **0.3** | 0.009112 | 0.106 7 | 15.5 | **0.004** | 3.4 | **0.3** |
| MCP1 | 0.001319 | 0.0113 0 | 0.001623 | 0.0173 6 | 1.2 | **>0.99** | 0.004540 | 0.040 64 | 3.4 | **0.5** | 2.8 | **0.3** |
| CX3CL1 | 9.089 e-007 | 0.0001 513 | 1.986 e-005 | 0.0019 35 | 21.8 | **0.7** | 0.000575 4 | 0.004 909 | 633 | **0.001** | 29.0 | **0.04** |
| CXCL10 | 1.712 e-005 | 0.0001 890 | 0.0001391 | 0.0025 28 | 8.1 | **0.6** | 4.996e-005 | 0.001 901 | 2.9 | **0.99** | 0.4 | **>0.99** |
| MX1 | 0.000196 4 | 0.0063 45 | 0.0003987 | 0.1728 | 2.0 | **0.7** | 0.03091 | 0.282 8 | 157.4 | **0.0007** | 77.5 | **0.02** |

**Table S2**: Medians and IQR values, comparisons (ratios) of the medians and exact P-values in SCAT

| | SIV- | | SIV+ | | | | SIV+ART+ | | | | | |
|---|---|---|---|---|---|---|---|---|---|---|---|---|
| | Medians | IQR | Medians | IQR | Ratio /SIV- | **p-value** | Medians | IQR | Ratio /SIV- | **p-value** | Ratio /SIV+ | **p-value** |
| PPARG | 0.004661 | 0.1634 | 0.003890 | 0.01885 | 0.8 | **0.9** | 0.02980 | 0.4765 | 6.4 | **0.2** | 7.7 | **0.003** |
| CEBPA | 0.0006882 | 0.9298 | 0.005331 | 0.04758 | 7.7 | **>0.99** | 0.009570 | 1.713 | 13.9 | **0.3** | 1.8 | **0.3** |
| COL1A2 | 0.001038 | 0.02399 | 0.001858 | 0.03243 | 1.8 | **>0.99** | 0.04915 | 0.6917 | 47.4 | **0.002** | 26.5 | **0.06** |
| PPARG/COL1A2 | 8.754 | 143.5 | 2.041 | 9.95 | 0.23 | **0.16** | 1.356 | 4.95 | 0.15 | **0.047** | 0.66 | **>0.999** |
| HIF1A | 0.006831 | 0.05820 | 0.001835 | 0.01594 | 0.3 | **>0.99** | 0.02686 | 0.2079 | 3.9 | **0.2** | 14.6 | **0.02** |
| CD45 | 6.977 e-006 | 0.0001055 | 2.658 e-006 | 0.0002276 | 0.4 | **>0.99** | 5.396 e-005 | 0.001356 | 7.7 | **0.3** | 20.3 | **0.09** |
| CD163 | 0.0006459 | 0.01120 | 0.0008414 | 0.005379 | 1.3 | **>0.99** | 0.002703 | 0.09786 | 4.2 | **0.1** | 3.2 | **0.04** |
| IFNG | 2.716 e-007 | 3.899 e-005 | 1.334 e-007 | 4.176 e-006 | 0.5 | **>0.99** | 2.902 e-005 | 0.002694 | 106.8 | **0.3** | 217.5 | **0.07** |
| IL6 | 0.0009372 | 0.02838 | 3.092 e-005 | 0.0006663 | 0.03 | **0.6** | 0.0003397 | 0.01102 | 0.4 | **>0.99** | 11.0 | **0.2** |
| TNFA | 1.711 e-007 | 1.156e-006 | 7.041 e-007 | 0.0003710 | 4.1 | **0.6** | 0.0001950 | 0.003503 | 1139.7 | **0.0003** | 277 | **0.01** |
| ADIPOQ | 0.03905 | 0.8469 | 0.04946 | 0.8327 | 1.3 | **>0.99** | 0.5703 | 5.889 | 14.6 | **0.047** | 11.5 | **0.07** |
| LEP | 0.02046 | 0.7438 | 0.06041 | 0.9938 | 3.0 | **>0.99** | 0.2285 | 1.345 | 11.2 | **0.1** | 3.8 | **0.4** |
| ADIPOQ/LEP | 0.5350 | 14.04 | 1.393 | 5.444 | 2.6 | **0.95** | 2.214 | 6.185 | 4.1 | **0.086** | 1.6 | **0.55** |
| CCL5 | 0.0007636 | 0.009266 | 0.001462 | 0.09206 | 1.9 | **0.8** | 0.01297 | 0.1020 | 17.0 | **0.02** | 8.9 | **0.2** |
| MCP1 | 0.0006122 | 0.02866 | 0.001179 | 0.003512 | 1.9 | **>0.99** | 0.005015 | 0.03005 | 8.2 | **0.2** | 4.3 | **0.1** |
| CX3CL1 | 7.656 e-006 | 0.0009774 | 4.607 e-005 | 0.0007388 | 6.0 | **>0.99** | 0.0002763 | 0.004876 | 36.1 | **0.04** | 6.0 | **0.08** |
| CXCL10 | 4.607 e-005 | 0.002371 | 7.238 e-005 | 0.003237 | 1.6 | **>0.99** | 0.0004344 | 0.01408 | 9.4 | **0.046** | 6.0 | **0.1** |
| MX1 | 0.0005093 | 0.02980 | 0.0007254 | 0.06549 | 1.4 | **>0.99** | 0.02082 | 0.4176 | 40.9 | **0.008** | 28.7 | **0.03** |

**Table S3:** Medians and IQR values, comparisons (ratios) of the medians and exact P-values in VAT
